# Socio-demographic determinants of electricity consumption across vietnamese households

**DOI:** 10.1371/journal.pone.0320758

**Published:** 2025-04-10

**Authors:** Duc Hong Vo

**Affiliations:** Research Centre in Business, Economics & Resources, Ho Chi Minh City Open University, Ho Chi Minh City, Vietnam; ECONOMIX, CNRS & University of Paris Nanterre, FRANCE

## Abstract

Reducing household energy consumption is generally considered a key force in limiting the adverse effects of human beings on climate change. As such, understanding the socio-demographic determinants of household electricity consumption across Vietnamese households plays an important role. However, this understanding has largely been neglected in the existing literature, particularly for Vietnam. This study focuses on understanding the role of socio-demographic factors electricity consumption across Vietnamese households using two waves of the Vietnamese Household Living Standards Surveys (VHLSS) in 2012 and 2020. The three-stage least squares estimation has been used to address the endogeneity problem and examines the reverse causality between income and electricity consumption of the households. The results show that household income and the educational level of the household head are the two factors with the most significant impacts on household electricity consumption. Electricity consumption expenditure also significantly and positively impacts household income. The economies of scale are confirmed between the number of household members and electricity consumption, meaning that larger families consume more electricity. In addition, our findings show that the differences in electricity consumption between the richest and poorest household groups increased sharply in 2020. These key findings call for policies to support disadvantaged households in Vietnam and that the current structure of the electricity tariff has failed to capture the disproportional level of electricity consumption between the richest and poorest groups of Vietnamese households.

## 1. Introduction

Energy plays an important role in the socio-economic development of countries across the world. Today, people have gradually switched from directly using traditional energy sources such as coal, firewood, and oil for their daily needs to secondary energy sources such as electricity. Electricity can come from fossil fuels, nuclear reactions, wind, solar and hydroelectric power [1,2]. Electricity has become the most important secondary energy source in household activities as it provides access to modern technology devices and helps people access information for education, work, and life quality in several ways [3]. Electrification has been shown to impact economic growth and improve the quality of life, health, and income in developing countries such as Vietnam [4] and Nepal [3].

With increased concerns about global climate change, pollution, and fossil fuel depletion; and the transition to environmentally friendly forms of energy taking place, energy consumption-related topics have received significant attention in recent years [5–7]. Many studies have consistently demonstrated that high energy consumption is a major driver of climate change [8], which, in turn, can harm energy security [9]. Energy consumption is found to negatively influence the environment by increasing CO2 emissions in both the short and long run [10–12]. Moreover, fossil fuel energy consumption tends to amplify the negative effects of financial inclusion on environmental sustainability, as increased energy access often leads to greater consumption of environmentally harmful energy sources [13]. As the world’s population and energy demand continue to grow, these negative environmental impacts are expected to intensify, with potentially catastrophic consequences for human well-being [14]. Furthermore, geopolitical tensions further complicate the issue of energy consumption and energy security. Current global conflicts and geopolitical instability make energy security a key issue in government policy [15]. The disruption of energy supply chains, driven by political conflicts and trade tensions, poses significant risks to both national and global energy security. In the context of rising climate change risk, fossil fuel depletion, and increasing energy supply disruptions, there is an urgent need for policymakers to better understand and manage energy consumption. Furthermore, current global conflicts and geopolitical instability make energy security a key issue in government policy [15]. Industry, trade, transport, and households are among the most important electricity consumers in the economy. Among those sectors, households play an important role in energy conservation and environmental protection [16,17]. Moreover, Han and Wei [18] consider that the residential sector consumes nearly one-third of the world’s total energy supply. Thus, energy policies aimed at the household sector should be a key concern for any government when they are serious about tackling the rising threat of climate change, the depletion of fossil fuels, and the increasing risk of energy supply disruptions.

Vietnam is one of the few developing countries with impressive economic growth in the past two decades and has the potential to become a “new tiger” of Asia. With the fast growth in recent years, the demand for energy has also increased rapidly [19]. As such, ensuring a stable energy supply is a focus of the Vietnamese government’s national development strategy. The country has been participating in trade agreements with many countries, regions, and territories as Vietnam continues integrating with the world’s economy [20]. Compliance with new strict environmental standards, the transformation to green energy has become one of the priorities. Over the past decade, this has also been the main driver of many of Vietnam’s solar and wind power projects. However, several issues should be considered with the accelerated development of renewable energy in Vietnam. For example, Vietnam’s national energy framework has now confirmed the state-led incentives to foster investment in local renewables deployment as the path to sustainability. Vietnam has committed to reducing heavy fossil fuel dependency to reach its net zero emissions target by 2050. The country has also expected that at least a third of Vietnam’s energy mix comes from renewable sources such as solar and wind. In particular, wind, solar and other renewable sources, excluding hydropower, are set to cover at least 31 per cent of the country’s energy needs by 2030 from 25 per cent in 2020 [21]. In contrast, hydropower would account for 19.5 per cent of the national energy mix in 2030, down from over 30 per cent in 2020. Coal would remain a crucial energy source, accounting for 20 per cent of the country’s energy mix by 2030. However, this level represents a significant reduction of this energy source from nearly 31 per cent in 2020 [21]. However, it is worth noting that renewable energy development throughout the national power system has faced the uncertainty of generation capacity depending on weather conditions and unstable power quality [22]. As such, in achieving the dual goals of ensuring energy security and switching to clean energy sources in the coming decades, it is necessary to have policies to encourage energy efficiency, in which the household sector has become an important policy target.

Previous studies have explored various factors influencing household energy consumption, including psychological and social factors [23], socio-demographic and physical characteristics, climate, and economic conditions [24–28]. In Vietnam, several studies have also investigated determinants of household electricity consumption, such as income, access to electricity, education, household size, and housing type [29], and price structure and heatwave shocks [30]. However, these studies rely on outdated datasets, which do not capture the recent near-universal access to electricity in the country [22]. Additionally, the research on the inequality in household electricity consumption across income levels is still limited. Furthermore, while the impact of energy use on economic growth has been studied at the macro level, few attempts have been made to examine the relationship between energy use and household income [3]. Consequently, it is necessary to conduct studies using up-to-date datasets to investigate factors influencing household electricity consumption in Vietnam. This study addresses this gap by employing the latest VHLSS survey data, alongside the 2012 VHLSS survey, to analyze patterns in household electricity consumption over the past decade.

In doing so, this study aims to address several key research questions: (i) What are the primary socio-demographic factors influencing household electricity consumption in Vietnam, given that access to electricity is nearly universal? (ii) Is there a non-linear relationship between household size and electricity consumption in Vietnam? and (iii) To what extent does income contribute to dispersion in electricity consumption across different income groups in Vietnam? This study is crucial in the current context as Vietnam has witnessed rapid economic growth and urbanization, which have led to increased energy demand and concerns about energy justice and environmental sustainability. As such, understanding determinants of household electricity consumption is essential for designing policies that promote energy savings, and equitable access to energy. Furthermore, with Vietnam’s commitment to addressing climate change and transitioning toward a greener economy, identifying patterns and disparities in electricity consumption can help policymakers design and implement more effective policies on electricity tariff.

This study contributes to the current literature in the following aspects. Firstly, this is the unique study that provides up-to-date statistics on household electricity consumption across all regions in Vietnam. These up-to-date statistics are critical for identifying regional disparities and trends that may inform targeted energy policies. Secondly, this study examines the influence of various socio-demographic factors on household electricity consumption in Vietnam, using the latest dataset. Thus, this study contributes to a deeper understanding of how socio-demographic changes affect energy demand. Thirdly, this study examines the bidirectional relationship between household income and electricity consumption using the three-stage least squares (3SLS) estimation approach. Fourthly, this study explores the non-linear relationship between household size and household electricity consumption, and the dispersion in household electricity consumption across income groups in Vietnam, which are limitedly studied in previous studies. By addressing these gaps, this study not only contributes to our understanding of household electricity consumption but also offers valuable insights and implications for policymakers in formulating effective energy policies.

The contributions of this study to the existing literature are twofold. *First*, this study exclusively focuses on understanding the determinants of electricity consumption across Vietnamese households, focusing on the socio-demographic characteristics of these major energy users. *Second*, empirical evidence from this study plays an important role in shaping appropriate public policy for Vietnam in the context of climate change, geopolitical risk, and political tensions among superpower nations globally to ensure energy security in supporting sustainable economic growth and social transformation in Vietnam.

Following the introduction, section 2 discusses relevant empirical studies related to energy usage. Section 3 provides details of the dataset and the procedure for constructing variables from the data. This section also introduces the methods used in the analysis. Section 4 presents and discusses empirical results using the ordinary least squares (OLS) and the three-stage least squares (3SLS) estimations, followed by the concluding remarks and policy implications in section 5 of the paper.

## 2. Literature review

In early research, theoretical models explaining household energy consumption draw on behavioral, socio-economic, and demographic perspectives. Van Raaij & Verhallen’s [28] introduce a behavioural model to explain the residual energy consumption. They examine the role of energy prices, socio-demographic factors, family lifestyle, energy-related behaviour, effectiveness and responsibility, cost-benefit trade-offs, feedback, information, and home characteristics. Costanzo et al. [23] discuss two groups of psychological and social factors that can influence the purchase behaviour of energy-saving appliances: psychological factors (i.e., perception, favourable evaluation, understanding, remembering) and positional/situational factors (i.e., disposable income, home ownership, home repair skills, own home technology), which help explain the decision-making process around energy-saving behaviors. While these models provide foundational insights, recent research expands on them to account for additional socio-demographic, and environmental factors that influence household energy consumption.

Different concepts have been used to identify and classify numerous interrelated factors influencing the variation of household energy consumption. The differences in energy consumption between households can be affected by many factors, including socio-demographic and physical characteristics, climate, and economic conditions [24–28,31–33].

In early research, Van Raaij and Verhallen [28] introduced a behavioural model to explain residual energy consumption. They examine the role of energy prices, socio-demographic factors, family lifestyle, energy-related behaviour, effectiveness and responsibility, cost-benefit trade-offs, feedback, information, and home characteristics. Costanzo et al. [23] discuss two groups of psychological and social factors that can influence the purchase behaviour of energy-saving appliances: psychological factors (i.e., perception, favourable evaluation, understanding, remembering) and positional/situational factors (i.e., disposable income, home ownership, home repair skills, own home technology).

Socio-demographic factors have a significant effect on household energy consumption behaviour. Results on household energy use related to socio-demographic aspects indicate that income, age, gender, household characteristics, dwelling characteristics and geographic location are the factors affecting energy usage [34–36]. Among these socio-demographic factors, income, education, and household size are the three important factors in household energy consumption patterns. First, income defines the budget constraint and influences the amount of energy a household can afford [31,36–39] . Second, education has a strong interrelationship with [40] and enables households to adopt new technologies and thus be more likely to purchase modern energy-using appliances. Higher education is also associated with the choice of cleaner and more efficient energy [36,41]. Third, household size is expected to increase energy use. However, the effect is often non-linear as household size increases; family members can share their energy-consuming appliances and thus use energy more efficiently [30,39]. Besides, there are several other factors influencing household electricity consumption including extreme temperatures [42], smart home adoption level [43], electrical household appliances, such as air conditioner, tumble dryer and deep freezer, [39], time-saving technology [44], green electricity tariffs [45]. Additionally, using provincial-level longitudinal data in China, Wang et al. [46] identify several key factors influencing urban household electricity consumption, including single-person households, floor area, urbanization rate, proportion of elderly individuals and per capita income.

Although different theoretical perspectives and models have emerged in the literature, selected models or approaches are widely accepted to explain household electricity usage comprehensively. No single approach has emerged as a good model for predicting differences in household electricity consumption. Current models often focus on explaining a particular aspect of the factors affecting household energy consumption behaviour. The empirical results show that the impacts of these factors on household electricity usage are different over time, context, participants and research topics [47].

In Vietnam, academic studies that focus on socio-demographic determinants are limited. Khandker et al. [48] examine the correlation between welfare outcomes and rural electrification, quantifying the benefits of electricity. The study uses panel surveys conducted from 2000 to 2005 among 1,120 rural households in 41 municipalities in Vietnam. The results indicate the positive effects of grid-connected electrification on household income, expenditure, and education. An early stage of electrification forms externalities that benefit the poor more than the rich, agriculture more than non-farm income, and girls more than boys in terms of schooling.

Son & Yoon [29] examine how the expansion of the national electricity grid has affected household electricity consumption over time using micro-data from the Vietnam Household Living Standards Survey from 1993 to 2004. The results show that household income is an important determinant of electricity consumption. This relationship is non-linear. The study also shows that electricity spending inequality is greater than income inequality. This result suggests that rapidly improving access to electricity may not directly lead to increased electricity use for low-income households and may widen the energy usage gap between high- and low-income households in an early stage of electrification. In addition, the level of education of the household heads, the household size and the type of housing are also important factors affecting electricity consumption.

Nguyen [30] examines the factors affecting household electricity demand in Vietnam. The study focuses on four factors: price structure, income, demographics, and heat waves. The study uses the 2012-2014-2016 VHLSS surveys to conduct a panel analysis. The results significantly affect the electricity poverty threshold between urban and rural areas. On the other hand, the effect of heat wave shocks is relatively small. The study examines the effects of demographic, economic, technical, and contextual factors on household electricity consumption in Vietnam. Furthermore, Khandker et al. [48] consider that electrification in Vietnam positively impacts Vietnamese households’ economy and health condition. Significant income inequality in Vietnam, which leads to a large discrepancy in electricity consumption between high-income and low-income households in the early stage of electrification, is also confirmed in Son & Yoon’s [29] study. As the proportion of households having access to the national electric grid has increased to over 95 per cent [22], the extent of energy inequality concerning electricity consumption needs to be re-examined.

Previous studies use outdated datasets to understand household energy demand patterns. In addition, the role of important factors in Vietnamese society, such as household characteristics, has largely been neglected. Furthermore, although the impact of energy use on economic growth has been examined at the macro level, only some attempts have been made to understand the relationship between energy use and family wealth [3]. As such, this study is conducted to achieve the following three objectives: (i) a better understanding of household energy patterns; (ii) a potential interrelationship between household income and electricity consumption expenditure; and (iii) the extent of the electricity consumption inequality between households of different income levels in Vietnam. Our study uses the latest VHLSS survey from 2020 and the 2012 VHLSS survey to examine the changes and trends in electricity consumption patterns in the past decade.

## 3. Methodology and data

### 3.1. Data

Our study uses two Vietnam Household Living Standard Surveys (VHLSS) in 2012 and 2020. Other information is collected from 63 Provincial Statistical Yearbooks of Vietnam. The consumer price index (CPI) is collected from the World Bank.

The 2020 VHLSS is the newest wave at the time this study was conducted, reflecting Vietnam’s current socio-economic conditions, including nearly universal access to electricity and rapid economic growth. This wave provides an up-to-date perspective on household energy consumption trends, particularly in the context of Vietnam’s commitments to green transitions and addressing climate change. The 2012 VHLSS, on the other hand, serves as a baseline, marking a period when Vietnam was still in the earlier stages of transitioning to a lower-middle-income economy and expanding its national electricity grid. Together, these two datasets allow for a decade-long comparative analysis, reflecting shifts in socio-demographic factors influencing electricity use and the inequality in electricity consumption across income groups.

The VHLSS is an important source of microdata on household well-being in Vietnam. It contains information reflecting the standard of living of Vietnamese households at the commune/ward and household levels. The survey covers people who do not work in Vietnamese government agencies. Household-level data collection from the VHLSS surveys includes the demographics of household members, educational background, income, expenditure, housing characteristics, sanitation, and anti-poverty program participation. At the commune/ward level, general information about living standards, infrastructure, production, and environmental conditions is collected [49].

The VHLSS data is divided into several subsections. A household income comes from many sources, including wages, subsidies, income from agriculture, forestry, fisheries, non-agricultural forestry and fisheries, and real estate rentals. As such, to calculate the total income of a household, the first step is to calculate the salary and allowances of each family member. Then, the total earnings from salary and allowances are combined to represent the household income. Next, in addition to working as wage earners, households can participate in many different production and business industries. As such, these sources of income are also included in the total household income. The income from production is calculated by subtracting the total costs from the total income from the goods produced and sold. The final step is to aggregate these directories based on each household’s identification number. The annual household income is finally determined. This calculation method is based on Vietnam’s General Statistics Office guidelines.

Total household electricity consumption is calculated by adding the annual total for electrical energy consumption for different purposes such as consumption (lighting, cooking, cooling) and commercial production (i.e., agriculture, forestry, fisheries, and other manufacturing and service industries). Household non-electricity consumption is also calculated. Household demographics are also aggregated based on each household’s identification number from the survey. Finally, monetary data are adjusted for the impact of inflation based on the CPI index compiled and reported by the World Bank. The sources and descriptions of the variables are provided in Table 1.

**Table 1:** Descriptions and sources of variables.

Variable	Description	Data Source
Electricity expenditure	Annual household total electricity expenditure (in log form) is calculated by summing the annual expenditures on electricity for various purposes, such as consumption (lighting, cooking, cooling) and commercial production (e.g., agriculture, forestry, fisheries, and other manufacturing and service industries).	VHLSSs 2012 & 2020
Household income	Household income is the sum of all income sources, including wages, subsidies, income from agriculture, forestry, fisheries, non-agricultural activities related to forestry and fisheries, and real estate rentals.	VHLSSs 2012 & 2020
Household size	The number of members in the household.	VHLSSs 2012 & 2020
Number of children	The number of children in the household	VHLSSs 2012 & 2020
Number of elders	The number of elders in the household	VHLSSs 2012 & 2020
Household head’s age	The age of the household head	VHLSSs 2012 & 2020
Female-headed households	1 if the household head is female, 0 otherwise.	VHLSSs 2012 & 2020
Education	The highest level of education attained by the household head.	VHLSSs 2012 & 2020
The total area of living room/bedroom	The total living and bedroom area of the house.	VHLSSs 2012 & 2020
Type of the house	The type of house in which the household resides	VHLSSs 2012 & 2020
Ownership of the house	The ownership status of the house in which the household resides	VHLSSs 2012 & 2020
Area	The region in which the household resides	VHLSSs 2012 & 2020

### 3.2. Methodology

We first examine the effect of socio-demographic factors on household electricity consumption, proxied by the annual electricity expenditure of the households, in Vietnam using the following estimation equation.


$$\begin{array}{c} lelectric_{i}=\beta_{0}+\beta_{1}Head_{i}+\beta_{2}HH_{i}+\beta_{3}D_{i}+\beta_{4}G_{i}+\varepsilon_{i} \end{array}$$ (1)

where lelec denotes the *annual total electricity expenditure* (in log form). HH is a vector of households’ characteristics such as *annual income* (in the log), *household size* and its square term; *Head* represents a vector of household head’s characteristics including *gender, education* (highest degree), *marriage status, age, and square of age*. *D* is a vector of dwelling characteristics, including *size* and *type of house*. *G* denotes the geographical information, including *location*, *rural/urban,* and *annual average temperature,* andε is the disturbance term.

The *total income* and *electricity expenditure* are log-transformed because they vary widely and exhibit outliers. In addition, few households do not spend any money on electricity, either because they cannot access the national grid or because they can self-supply electricity. However, these households account for a very small proportion. As such, they are excluded from the final sample. In our sample, the number of households that do not spend on electricity was approximately 2 per cent in 2012 and 1 per cent in 2020 of the total sample size. Also, several dummies representing different income deciles are added to various regression equations to examine the energy dispersion/inequality level regarding household income.

We use a similar approach to Bridge et al. [37] to examine the potential effect of electricity consumption on household income. We assume there is an endogeneity of income based on the findings of previous studies [3,37,48,50]. Since household members in Vietnam can be involved in many different industries at the same time, electricity plays an important role as an input for household production and business. As such, access to electricity helps households have an efficient and cheap energy source to produce products and services and, hence, have more income. In addition, electricity usage in households also helps improve the quality of life of the household members and thereby positively affects the working capacity and productivity of family members [3,14]. Using the house ownership status, the house’s total living/bedroom area is the exogenous variable in the system of equations. We estimate a system of two simultaneous equations representing the reverse causality between *electricity expenditure* and *consumption*. The three-stage least squares (3SLS) method, first introduced by Zellner [51], is used. This 3SLS combines the seemingly unrelated regression methods (SUR) and the two-stage least squares method (2SLS).

The following system of equations is estimated:


$$\{\begin{array}{c} lelectric_{i}=\beta_{0}+\beta_{1}lconsump_{i}+\beta_{2}floor_{i}+\beta_{3}roof_{i}+\beta_{4}\sum Z_{i}+\varepsilon_{i}\\ lconsump_{i}=\beta_{5}+\beta_{6}lelectric_{i}+\beta_{7}ownership_{i}+\beta_{8}\sum Z_{i}+\varepsilon_{i} \end{array}$$ (2,3)

Endogenous variables: *lelectric*_*i*_*, lconsump*_*i*_

Excluded exogenous variables: *floor*_*i*_*, roof*_*i*_*, ownership*_*i*_

*floor*_*i*_ denotes the total area of the living room and bedrooms, *roof*_*i*_ represents the roof type of the house. *ownership*_*i*_ stands for the ownership status of the house. Z_i_ denotes the vector of other exogenous variables in both equations, including gender, education, household size, head’s marital status, head’s age and its square, rural/urban area, and the number of children and elders. The expenditure on electricity was subtracted from the total consumption of the household.

The excluded exogenous variables in our analysis are house characteristics (total area of living rooms and bedrooms, and the roof type of the house) and house ownership status. These variables were selected because they are expected to influence household electricity consumption but not directly affect household income. On the one hand, the total area of living rooms and bedrooms and the roof type of the house are directly relevant to household electricity consumption because they represent structural characteristics of the household environment. Larger living spaces typically require more electricity for lighting, heating, cooling, and appliances. Similarly, the roof type reflects insulation quality, which significantly affect energy usage for maintaining indoor temperature. Thus, the total area of living rooms and bedrooms and the roof type of the house can affect household electricity consumption, thereby influencing household income. On the other hand, the total area of living rooms and bedrooms and the roof type of the house cannot directly influence income-generating activities —or at least, there is little evidence of this in the current literature. Regarding the ownership status of the house, it is directly relevant to household income. While homeowners may make decisions that affect electricity consumption (e.g., investing in energy-efficient appliances), these decisions are mediated by household income. Ownership status itself does not causally determine electricity consumption in the absence of household income effects. As such, the ownership status of the house is a valid excluded variable for the electricity consumption equation.

The three-stage least squares (3SLS) method is particularly advantageous in contexts where multiple equations are interdependent and there is potential endogeneity between variables across these equations. Indeed, 3SLS has been widely utilized in the literature [3,37,52,53]. Unlike the two-stage least squares (2SLS) method, which addresses endogeneity within a single equation, 3SLS combines the seemingly unrelated regression (SUR) method with 2SLS and simultaneously estimates all equations in a system. This combination enables 3SLS to exploit both cross-equation error correlation and the instrumental variables to address endogeneity, particularly reverse causality between variables (as in our study of income and electricity consumption). As such, 3SLS reduces the risk of biased estimates that could arise if errors in the equations were assumed to be uncorrelated. However, it should be noted that 3SLS is more sensitive to specification errors than 2SLS, as errors in one equation can propagate across other equations when all are estimated simultaneously. In contrast, a key advantage of 2SLS is its robustness to model misspecification. To mitigate the risk of misspecification, we take the natural logarithm of both household electricity consumption and household income. Additionally, given the theoretical and empirical support discussed above, we consider 3SLS to be an appropriate method for our analysis.

## 4. Empirical results on electricity consumption and household income

### 4.1. The descriptive statistics

The data from VHLSS 2012 has a total of 8,790 observations. The average annual income of households is approximately VND 150,000,000 (approximately US$ 7,200) in 2012. The average number of members of each household is approximately 4 persons, and the average age of the household’s head is 49. The data from VHLSS 2020 has a total of 8,573 observations. The average annual income of households is approximately VND 260,000,000 (approximately US$ 11,100) in 2020. From these two surveys, the average household income increased by approximately 73 per cent. However, the average household electricity expenditure increased from approximately VND 2,657,000 in 2012 to VND 6,547,000 in 2020 to VND 6,547,000 in 2020, representing almost 150 per cent. This significant increase in household electricity expenditure aligns with significant economic achievement when Vietnam officially became a lower-middle-income country in 2011, according to the United Nations Development Programme report. The average number of members of each household is approximately four people, and the average age of the household’s head is 50. Table 2 below presents descriptive statistics of the variables used in the analysis.

**Table 2:** Descriptive statistics – VHLSS 2012 versus VHLSS 2020 surveys.

Variables	Obs.	Mean	Std. Dev.	Min	Max
**VHLSS 2012**					
Electricity expenditure	8,790	2,657	8,042	0	487,200
Household income	8,787	149,617	328,019	1830	15,114,599
Household size	8,790	3.875	1.518	1	12
Number of children	8,790	.908	.967	0	6
Number of elders	8,790	.476	.725	0	4
Household head’s age	8,790	49.3	13.8	13	95
Square of household head’s age	8,790	2,628	1,477	169	9,025
Female-headed households	8,790	1.237	0.425	1	2
Education	8,790	2.605	1.163	1	5
The total area of living room/bedroom	8,783	75.9	47	6	608
Type of the house	8,783	3.607	0.886	1	5
Ownership of the house	8,783	1.977	0.149	1	2
Area	8,790	3.291	1.817	1	6
**VHLSS 2020**					
Electricity expenditure	8,573	6,547	22,831	0	1,468,800
Household income	8,573	260,719	549,213	7,200	34,782,300
Household size	8,573	3.677	1.548	1	12
Number of children	8,573	.94	1.001	0	6
Number of elders	8,573	.581	.787	0	4
Household head’s age	8,573	50.807	13.784	17	99
Square of household head’s age	8,573	2,771	1,491	289	9,801
Female-headed households	8,573	1.253	.435	1	2
Education	8,573	2.818	1.169	1	5
The total area of living room/bedroom	8,570	94.8	59.25	6	820
Type of the house	8,570	3.288	0.979	1	5
Ownership of the house	8,570	1.965	0.184	1	2
Area	8,573	3.219	1.803	1	6

Source: VHLSS 2012 & VHLSS 2020

*Notes:* The average nominal exchange rates between VND and USD were approximately US$ 1 = VND 23,412 (for 2020) and US$ 1 = VND 20,857. The rates are available at https://www.exchange-rates.org/exchange-rate-history/usd-vnd-2012. Accessed on 12 August 2024.

Table 3 summarizes Vietnamese households and household heads in 2012 and 2020. Compared to 2012, the income level of Vietnamese households has significantly increased. For example, the number of households with an income lower than VND 30 million per year decreased from 792 households in 2012–192 in 2020.

**Table 3:** The summary of household characteristics.

Variables	2012			2020		
		***Observations***	***Per cent***		***Observations***	***Per cent***
*Gender (household head)*	Male Female	6,706 2,084	76.29 23.71	Male Female	6,405 2,168	74.71 25.29
*Education* *(Household head)*	No degree	1,791	20.38	No degree	1,266	14.77
	Primary school	2,412	27.44	Primary school	2,208	25.76
	Lower secondary school	2,641	30.05	Lower secondary school	2,695	31.44
	Upper secondary school	1,370	15.59	Upper secondary school	1,630	19.01
	College or above	576	6.55	College or above	774	9.03
*Household income (million Vietnam Dong)*	<30 [30,100] [100,300] >300	792 3923 3322 753	9.01 44.63 37.79 8.57	<30 [30,100] [100,300] >300	192 1,767 4,667 1,947	2.24 20.61 54.44 22.71
*Household size*	[1,2] [3,4] [5,6] >6	1,666 4,519 2,915 410	18.95 51.41 24.95 4.66	[1,2] [3,4] [5,6] >6	2,149 4,129 1,975 320	25.07 48.16 23.04 3.73

Source: *VHLSS 2012 and VHLSS 2020*

*Notes:* The average nominal exchange rates between VND and USD were approximately US$ 1 = VND 23,412 (for 2020) and US$ 1 = VND 20,857. The rates are available at https://www.exchange-rates.org/exchange-rate-history/usd-vnd-2012. Accessed on 12 August 2024.

**Fig 1:**
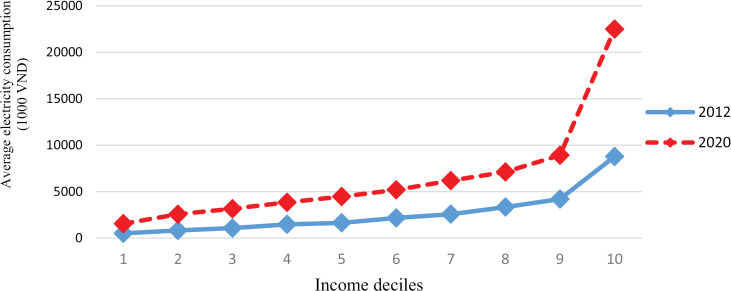
Average annual electricity consumption by income deciles.

Fig 1 shows the average annual electricity consumption differences by income deciles. The average electricity consumption of households with the highest income decile increased sharply in 2020 compared to 2012. As a result, the gap between the highest and lowest-income family groups has widened rapidly.

**Fig 2:**
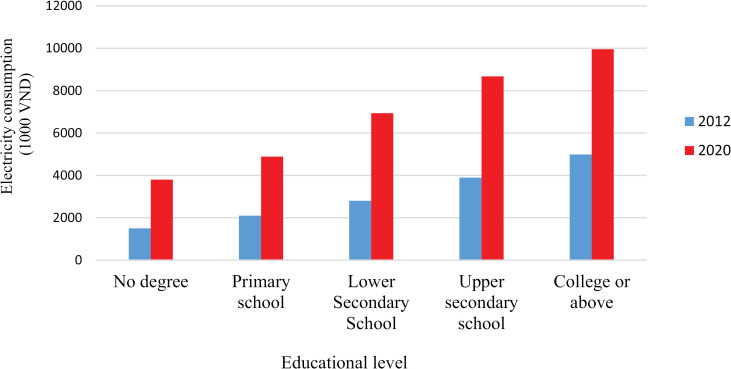
Average annual electricity consumption by educational level of the household heads.

Fig 2 shows the average electricity expenditure by a group of household heads with different educational levels. The higher the household head’s educational level, the more electricity the household consumes.

The Red River Delta region (including the capital city Ha Noi) and the Southeast region (including the largest city in Vietnam) are the two areas with the highest average household electricity consumption. Conversely, the Central Highlands and the Northern mountainous regions have relatively low electricity consumption growth rates.

### 4.2. Estimation results

#### 4.2.1. The OLS estimation.

First, we estimate Equation (1), as specified in subsection 3.2, using OLS estimation. The model’s dependent variable is the household’s total electricity expenditure within a year. The main explanatory variables include income, number of members in the household, and place of residence. Our study also considers the demographic characteristics of the household head, such as education level, age, gender, and marital status. This study uses the traditional approach of only one equation to explain household energy demand. In addition, the 3SLS approach is also applied to investigate the possible two-way relationship between electricity consumption and household income. This estimation method also helps solve the potential endogeneity problem that occurs in the model because of this reverse causality. Empirical results for the baseline model are presented in Table 4 below.

**Table 4:** The determinants of household electricity expenditure (OLS estimation).

Log(electric)	2012			2020		
	Model 1	Model 2	Model 3	Model 1	Model 2	Model 3
Variables	OLS	OLS	OLS	OLS	OLS	OLS
Log(income)		0.605^***^			0.574^***^	
		(0.012)			(0.013)	
2^nd^ income decile			0.343^***^			0.419^***^
			(0.034)			(0.031)
3^rd^ income decile			0.516^***^			0.590^***^
			(0.034)			(0.031)
4^th^ income decile			0.737^***^			0.748^***^
			(0.035)			(0.032)
5^th^ income decile			0.815^***^			0.852^***^
			(0.035)			(0.033)
6^th^ income decile			0.989^***^			0.923^***^
			(0.036)			(0.033)
7^th^ income decile			1.091^***^			1.053^***^
			(0.036)			(0.034)
8^th^ income decile			1.268^***^			1.132^***^
			(0.037)			(0.034)
9^th^ income decile			1.438^***^			1.326^***^
			(0.037)			(0.035)
10^th^ income decile			1.875^***^			1.815^***^
			(0.038)			(0.035)
Number of Members	0.380^***^	0.158^***^	0.158^***^	0.435^***^	0.185^***^	0.194^***^
	(0.022)	(0.020)	(0.020)	(0.020)	(0.019)	(0.018)
Square of the number of family members	−0.026^***^	−0.014^***^	−0.014^***^	−0.033^***^	−0.017^***^	−0.019^***^
	(0.002)	(0.002)	(0.002)	(0.002)	(0.002)	(0.002)
**Area**						
Northern midland and mountainous	−0.385^***^	−0.261^***^	−0.258^***^	−0.442^***^	−0.262^***^	−0.284^***^
	(0.029)	(0.026)	(0.025)	(0.026)	(0.024)	(0.022)
Central Coastal	−0.130^***^	−0.077^***^	−0.080^***^	−0.295^***^	−0.222^***^	−0.244^***^
	(0.026)	(0.023)	(0.022)	(0.024)	(0.022)	(0.021)
Central highland	−0.011	−0.029	−0.089^***^	−0.439^***^	−0.333^***^	−0.418^***^
	(0.039)	(0.034)	(0.033)	(0.036)	(0.032)	(0.030)
Southeast	0.462^***^	0.206^***^	0.242^***^	0.183^***^	0.027	0.019
	(0.032)	(0.029)	(0.027)	(0.031)	(0.028)	(0.027)
Mekong river delta	0.175^***^	0.051^*^	0.018	0.007	−0.079^***^	−0.122^***^
	(0.030)	(0.026)	(0.025)	(0.028)	(0.025)	(0.024)
**Households in the rural area**	−0.444^***^	−0.281^***^	−0.320^***^	−0.282^***^	−0.190^***^	−0.211^***^
	(0.021)	(0.019)	(0.018)	(0.018)	(0.017)	(0.016)
**Average temperature**	0.084^***^	0.066^***^	0.062^***^	0.071^***^	0.070^***^	0.053^***^
	(0.008)	(0.007)	(0.007)	(0.009)	(0.008)	(0.008)
**House type**						
House type = Permanent w/private bath	−0.178^*^	−0.144	−0.147	−0.160^*^	−0.097	−0.078
	(0.105)	(0.093)	(0.089)	(0.085)	(0.076)	(0.073)
House type = Permanent w/shared bath	−0.601^***^	−0.412^***^	−0.421^***^	−0.408^***^	−0.244^***^	−0.214^***^
	(0.107)	(0.094)	(0.090)	(0.087)	(0.078)	(0.075)
House type = Semi-permanent	−0.754^***^	−0.489^***^	−0.494^***^	−0.606^***^	−0.360^***^	−0.327^***^
	(0.105)	(0.093)	(0.089)	(0.085)	(0.077)	(0.073)
House type = Temporary	−1.187^***^	−0.725^***^	−0.716^***^	−1.026^***^	−0.607^***^	−0.555^***^
	(0.109)	(0.097)	(0.093)	(0.093)	(0.084)	(0.080)
**Educational level**						
Primary school	0.264^***^	0.166^***^	0.150^***^	0.186^***^	0.131^***^	0.131^***^
	(0.026)	(0.023)	(0.022)	(0.026)	(0.023)	(0.022)
Lower Secondary School	0.419^***^	0.247^***^	0.235^***^	0.324^***^	0.203^***^	0.195^***^
	(0.027)	(0.024)	(0.023)	(0.026)	(0.024)	(0.023)
Upper secondary school	0.597^***^	0.339^***^	0.341^***^	0.516^***^	0.326^***^	0.321^***^
	(0.031)	(0.028)	(0.027)	(0.029)	(0.027)	(0.026)
College or above	0.647^***^	0.217^***^	0.298^***^	0.596^***^	0.252^***^	0.303^***^
	(0.042)	(0.038)	(0.036)	(0.036)	(0.033)	(0.032)
**Age**						
Age of Head	0.043^***^	0.022^***^	0.017^***^	0.058^***^	0.036^***^	0.032^***^
	(0.004)	(0.003)	(0.003)	(0.004)	(0.003)	(0.003)
Square of head’s age	−0.000^***^	−0.000^***^	−0.000^***^	−0.000^***^	−0.000^***^	−0.000^***^
	(0.000)	(0.000)	(0.000)	(0.000)	(0.000)	(0.000)
**Marital status**						
Head is married	0.346^***^	0.215^***^	0.221^***^	0.264^***^	0.196^***^	0.185^***^
	(0.057)	(0.051)	(0.049)	(0.050)	(0.045)	(0.043)
Head is widowed	0.199^***^	0.162^***^	0.154^***^	0.145^***^	0.137^***^	0.124^***^
	(0.060)	(0.053)	(0.051)	(0.054)	(0.049)	(0.046)
Head is divorced	0.251^***^	0.225^***^	0.213^***^	0.174^***^	0.149^***^	0.178^***^
	(0.081)	(0.072)	(0.069)	(0.064)	(0.057)	(0.055)
Head is separated	0.205^*^	0.178^*^	0.213^**^	−0.044	−0.102	−0.085
	(0.110)	(0.097)	(0.094)	(0.107)	(0.096)	(0.092)
Head is female	0.041	0.011	0.047^**^	0.055^**^	0.043^**^	0.062^***^
	(0.026)	(0.023)	(0.022)	(0.022)	(0.020)	(0.019)
Constant	5.191^***^	−0.257	5.581^***^	5.337^***^	−0.053	5.679^***^
	(0.159)	(0.179)	(0.135)	(0.137)	(0.172)	(0.118)
Observations	8,587	8,583	8,584	8,531	8,524	8,531
R-squared	0.407	0.537	0.574	0.394	0.510	0.557

Standard errors in parentheses

*** p<0.01,

** p<0.05,

* p<0.1

In model 1, the *income* variable is not included. Income, education, and the type of house the family lives in have a very close relationship. Income impacts total electricity consumption, educational level, and house type. This study examines the effects of other socio-demographics on electricity consumption when income is included in the disturbance term. In models 2 and 3, income variables are included to consider how the results change.

Our empirical results indicate that when income is added to the models, the estimated coefficients in model 1 change significantly. However, income strongly correlates with education level and the type of house in which the family lives, so the problem of multicollinearity is unavoidable. However, the sign and magnitude of the estimated coefficients in all models indicate that a household’s total income strongly correlates with the household’s total electricity consumption. Model 3, including ten different income deciles, produces statistically significant results - the higher the decile a family falls into, the higher the household will consume electricity. Compared to households in the 1^st^ decile, the average households in the 10^th^ decile consumed more than 552 per cent (the estimated coefficient is 1.875) in 2012 and more than 514 per cent (1.815) in 2020. This result shows a profound level of energy consumption dispersion in Vietnam. This finding is consistent with the results from Khandker et al. [50], where the income poor is also the energy poor. Many studies have shown that middle-income households are the most energy-efficient group, unlike low-income households that cannot afford energy-efficient and high-income households that are reluctant to reduce usage [29].

The positive impact of income on electricity consumption can be explained as follows. Higher-income households tend to live in larger homes with more rooms, higher ceilings, and additional amenities (e.g., swimming pools, home offices). Larger homes require more energy for heating, cooling, and lighting. Moreover, high-income households are more likely to own second homes or vacation properties, which further increases total energy use. Besides, the ‘energy ladder’ hypothesis provides another explanation for the link between income and electricity consumption. As households move up the income ladder, they tend to shift from traditional, less convenient energy sources (such as biomass or coal) to more efficient and convenient forms like electricity. Higher-income households are more likely to fully transition to electricity, not only for lighting but also for cooking, heating, and other household activities. This shift is driven by a preference for the cleanliness, convenience, and efficiency of electricity compared to alternative fuels. Finally, while higher-income households are more likely to invest in energy-efficient appliances, this does not necessarily translate into lower electricity consumption. This phenomenon, known as the “energy efficiency paradox” or the “rebound effect,” occurs when the lower operating costs of efficient appliances lead to more frequent use, negating potential energy savings. In this way, higher income not only facilitates the purchase of efficient appliances but may also drive an increase in overall electricity demand. Indeed, Zheng et al. [31] find that energy efficiency is lowest in the high-income group, where it declines rapidly. Meanwhile, the low-income group has the highest energy efficiency, where it declines slowly.

**Fig 3:**
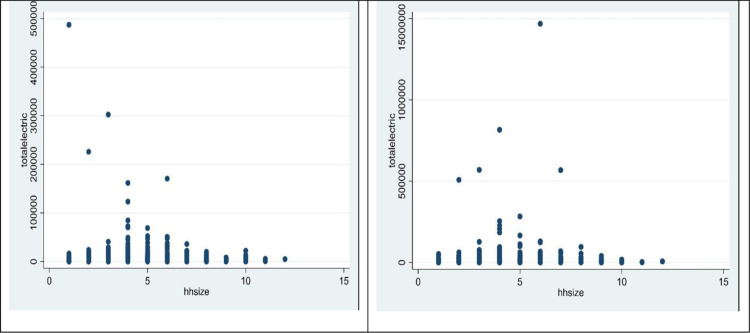
Scatter plot between household size and electricity consumption – 2012 and 2020.

The positive sign of the estimated coefficients of the *number of members* and the negative sign of the coefficient of the *square of the number of members* shows that the economics of scale in household electricity use exists. When the initial household member increases by one person, the household’s total electricity consumption will increase. However, when people are large enough, the shared use of many electrical appliances will reduce the total electricity consumption. This economics of scale phenomenon can be observed directly in Figs 1 and 3. In both years, the total electricity expenditure decreased when the household size exceeded five members. These results are similar to those of Ironmonger et al. [54] and Nguyen [30].

The geographical location in which the family lives is also correlated with the household’s electricity consumption. For example, compared with the Red River Delta region, households in the Southeast region consume more electricity. This finding is observed in the results for 2020. Households living in the Central Coastal, Northern Central Highlands, and Northern Midlands and Mountains regions will have lower total electricity consumption. Compared to households in the Red River Delta region, the average household in the Mekong Delta region consumed more electricity in 2012 but less in 2020.

The type of house a household lives in also significantly impacts electricity consumption. Households live in temporary or semi-permanent houses; on average, semi-permanent houses usually have lower total electricity consumption than families living in permanent houses with private baths and villas. This is because houses with modern designs often integrate many lighting devices and other power-consuming devices. The average temperature of the province where the household stays also shows a positive relationship with electricity expenditure.

Next, the household head’s education level also strongly impacts the household’s total electricity consumption, even when controlling for income level. Compared with household heads without professional qualifications, household heads with a high school or college education correlate with 43–50 per cent more total electricity consumption. An explanation for the strong influence of education on household electricity consumption is that education can increase awareness and familiarity with modern technological advancements, which can drive households to adopt modern technology, appliances and devices. These devices, while enhancing convenience and lifestyle quality, typically require significant electricity usage, thereby increasing overall household energy consumption. Furthermore, education also influences household energy choices, particularly the preference for cleaner and more efficient energy sources like electricity [55]. Educated individuals are more likely to be aware of the environmental and health benefits of using electricity compared to traditional energy sources, such as biomass (e.g., wood, charcoal) or coal. As a result, households whose head has higher level of education are more likely to choose to use electricity for cooking, heating, and lighting instead of relying on polluting alternatives. Although this shift supports cleaner energy consumption, it also leads to higher electricity usage, as electric appliances often replace less efficient but less energy-intensive traditional practices. However, the reason could be that access to education makes it easier for homeowners to access new technology and purchase various devices.

The coefficients of the variables related to the age of the household head are also similar to those of previous studies. Total electricity consumption will increase with age, but as people get older, their electricity use will decrease. The coefficients of the variables representing this relationship are small because most of the sample household heads are over 40 years old. This finding suggests that as initially the age of the household head increases, total electricity consumption tends to rise. This can be attributed to higher income levels, larger household sizes, and a greater desire for comfort and convenience during middle age. However, beyond a certain age, electricity usage typically declines as household heads retire, household sizes shrink (e.g., children move out), and lifestyle preferences shift toward less energy-intensive activities. Finally, when compared with unmarried householders, married household heads have a higher correlation with total household electricity consumption. This finding may be due to the fact that married households often have larger family sizes, leading to greater overall energy needs for activities such as cooking, heating, and appliance usage. Additionally, married couples may place a higher emphasis on creating a comfortable home environment, resulting in increased usage of energy-consuming devices like air conditioners, washing machines, and entertainment systems. This preference for comfort and shared household activities contributes to higher electricity consumption compared to unmarried households, who may have simpler living arrangements and fewer energy-intensive appliances. One interesting finding is that if a family’s head is female, then the total electricity expenditure of that family will be higher than that of a male. Households headed by women who are divorced also have higher total electricity consumption. This difference can be explained by the fact that many electricity-consuming activities, such as cooking and washing, are more often carried out by women, which may lead to higher overall energy needs in female-headed households [56].

Household energy consumption is affected by multiple interrelated factors. In our model, household income may be correlated with many other explanatory variables such as education, type of house, age, and home location. We show how important income is as it affects households’ behaviour in consuming electricity and slightly influences other factors in the model. However, the degree of multicollinearity is small and not an important problem since all the statistically significant coefficients in model 1 remain statistically significant in models 2 and 3 at the same significant levels. Including income in the model helps considerably increase the value of the R-square.

The effects of income, household size, house type, marital status of the householder, and educational level are relatively consistent and stable between 2012 and 2020. Moreover, the negative coefficients, which represent the relative differences between other areas compared to the Red River Delta region, have decreased even more in 2020, suggesting that household energy consumption in the Red River Delta region has increased higher than in other regions.

In the second analysis, household income, household size, and types of houses are added to the model to examine the robustness of the results. A large number of families in Vietnam participate in multiple sectors at the same time, and many of those are informal markets. Therefore, the income estimation from these sources needs to be more accurate. Thus, another way to estimate household income is by measuring consumption per capita. The annual household income is substituted by household non-electricity consumption per capita and its deciles. In addition, the type of dwelling is also substituted by the type of roof and wall. Finally, several dummy variables representing different household sizes replaced the total number of household members.

As Table 5 shows, the estimation results have largely remained unchanged compared to those from the baseline models. Our empirical results presented and discussed in this study are robust across different proxies.

**Table 5:** The robustness test.

	2012		2020	
	Model 1	Model 2	Model 1	Model 2
Variables	OLS	OLS	OLS	OLS
Log(consumption)	0.365^***^		0.608^***^	
	(0.016)		(0.015)	
2^nd^ consumption decile		0.151^***^		0.297^***^
		(0.039)		(0.033)
3^rd^ consumption decile		0.215^***^		0.515^***^
		(0.040)		(0.034)
4^th^ consumption decile		0.275^***^		0.634^***^
		(0.041)		(0.035)
5^th^ consumption decile		0.338^***^		0.759^***^
		(0.041)		(0.036)
6^th^ consumption decile		0.458^***^		0.835^***^
		(0.042)		(0.036)
7^th^ consumption decile		0.519^***^		0.977^***^
		(0.042)		(0.037)
8^th^ consumption decile		0.572^***^		1.019^***^
		(0.043)		(0.038)
9^th^ consumption decile		0.668^***^		1.148^***^
		(0.044)		(0.039)
10^th^ consumption decile		0.840^***^		1.357^***^
		(0.045)		(0.041)
Household with 1 member	−1.260^***^	−0.689^***^	−1.499^***^	−0.436^***^
	(0.056)	(0.062)	(0.048)	(0.053)
Household with 2 members	−0.790^***^	−0.365^***^	−0.800^***^	−0.108^***^
	(0.039)	(0.044)	(0.036)	(0.040)
Household with 3 members	−0.468^***^	−0.184^***^	−0.516^***^	−0.078^**^
	(0.036)	(0.038)	(0.034)	(0.036)
Household with 4 members	−0.299^***^	−0.123^***^	−0.315^***^	−0.052^*^
	(0.031)	(0.032)	(0.030)	(0.030)
Household with 5 members	−0.214^***^	−0.117^***^	−0.159^***^	−0.017
	(0.031)	(0.032)	(0.029)	(0.029)
The household has 1 child	−0.059^**^	−0.056^**^	−0.055^**^	−0.054^**^
	(0.023)	(0.023)	(0.022)	(0.022)
The household has 2 children	0.070^**^	0.070^**^	0.066^**^	0.060^**^
	(0.029)	(0.029)	(0.027)	(0.027)
The household has 3 or more children	−0.023	−0.014	0.044	0.017
	(0.047)	(0.047)	(0.041)	(0.041)
The household has 1 elder	0.031	0.028	0.004	0.000
	(0.025)	(0.025)	(0.022)	(0.022)
The household has two or more elders	−0.023	−0.023	−0.037	−0.026
	(0.035)	(0.035)	(0.027)	(0.027)
Northern midland and mountainous	−0.208^***^	−0.204^***^	−0.214^***^	−0.216^***^
	(0.030)	(0.030)	(0.025)	(0.025)
Central Coastal	−0.101^***^	−0.099^***^	−0.291^***^	−0.288^***^
	(0.027)	(0.027)	(0.023)	(0.023)
Central highland	0.040	0.045	−0.345^***^	−0.343^***^
	(0.041)	(0.041)	(0.035)	(0.035)
Southeast	0.426^***^	0.440^***^	0.047	0.059^*^
	(0.035)	(0.035)	(0.031)	(0.031)
Mekong river delta	0.192^***^	0.205^***^	−0.005	−0.000
	(0.034)	(0.034)	(0.029)	(0.029)
Households in rural areas	−0.369^***^	−0.373^***^	−0.154^***^	−0.168^***^
	(0.020)	(0.020)	(0.017)	(0.017)
Tile roof	−0.350^***^	−0.354^***^	−0.221^***^	−0.234^***^
	(0.027)	(0.027)	(0.023)	(0.023)
Sheet roof	−0.283^***^	−0.292^***^	−0.273^***^	−0.286^***^
	(0.030)	(0.030)	(0.023)	(0.023)
Thatch roof	−0.378^***^	−0.388^***^	−0.105	−0.120
	(0.060)	(0.061)	(0.079)	(0.079)
Other types of roof	−0.155	−0.152	−0.285	−0.397
	(0.221)	(0.222)	(0.250)	(0.251)
Brick, stone wall	0.014	0.012	−0.038	−0.042
	(0.052)	(0.052)	(0.035)	(0.035)
Wooden/metal wall	−0.385^***^	−0.377^***^	−0.446^***^	−0.443^***^
	(0.059)	(0.059)	(0.044)	(0.044)
Soil/straw bale wall	−0.339^***^	−0.327^***^	−0.336^***^	−0.349^***^
	(0.078)	(0.079)	(0.105)	(0.106)
Plywood wall	−0.550^***^	−0.552^***^	−0.479^***^	−0.463^***^
	(0.070)	(0.071)	(0.078)	(0.079)
Other wall types	−0.476^***^	−0.476^***^	−0.414^***^	−0.397^***^
	(0.071)	(0.072)	(0.072)	(0.072)
Primary school	0.211^***^	0.215^***^	0.125^***^	0.131^***^
	(0.025)	(0.025)	(0.024)	(0.024)
Lower Secondary School	0.321^***^	0.329^***^	0.202^***^	0.212^***^
	(0.026)	(0.026)	(0.024)	(0.024)
Upper secondary school	0.469^***^	0.475^***^	0.324^***^	0.336^***^
	(0.031)	(0.031)	(0.027)	(0.027)
College or above	0.508^***^	0.536^***^	0.270^***^	0.303^***^
	(0.041)	(0.041)	(0.034)	(0.034)
Age of Head	0.033^***^	0.033^***^	0.040^***^	0.041^***^
	(0.004)	(0.004)	(0.003)	(0.003)
Square of age	−0.000^***^	−0.000^***^	−0.000^***^	−0.000^***^
	(0.000)	(0.000)	(0.000)	(0.000)
Head is married	0.302^***^	0.271^***^	0.164^***^	0.181^***^
	(0.057)	(0.058)	(0.047)	(0.047)
Head is widowed	0.161^***^	0.136^**^	0.088^*^	0.107^**^
	(0.061)	(0.061)	(0.051)	(0.051)
Head is divorced	0.226^***^	0.198^**^	0.097^*^	0.124^**^
	(0.079)	(0.080)	(0.059)	(0.059)
Head is separated	0.217^**^	0.206^*^	−0.127	−0.086
	(0.107)	(0.107)	(0.098)	(0.098)
Head is female	0.016	0.007	0.041^**^	0.040^*^
	(0.025)	(0.025)	(0.020)	(0.021)
Constant	3.236^***^	5.896^***^	1.202^***^	6.036^***^
	(0.199)	(0.147)	(0.190)	(0.123)
Observations	8,574	8,587	8,531	8,531
R-squared	0.447	0.445	0.502	0.498

Standard errors in parentheses

*** p<0.01,

** p<0.05,

* p<0.1

#### 4.2.2. Empirical results on electricity consumption using the three-stage least square estimation.

We now use the three-stage least square (3SLS) estimation to examine the effects of electricity consumption on household income, which is proxied by household consumption. Equations (2) and (3) are used to perform the 3SLS estimation. We notice that compared to the first estimation using OLS, the estimated coefficients of log(consume) increased from 0.37 to 0.42 in 2012 and from 0.61 to 0.46 in 2020 after applying the 3SLS method. All these estimates are positive and significant at the 1 per cent level. The complete results using the three-stage least square are presented in Table 6. This finding implies that income still significantly affects electricity expenditure even when we have accounted for the endogeneity issue. This result is also similar to the finding of Son & Yoon [29], indicating that households with higher incomes are more likely to buy several electrical appliances that consume more electricity and, therefore, face higher electric bills.

**Table 6:** The effects of income on electricity expenditure using the three-stage least squares estimations.

	2012		2020	
	(3SLS)		(3SLS)	
Variables	Log(consumption)	Log(electric)	Log(consumption)	Log(electric)
Log(consumption)		1.096^***^		0.733^***^
		(0.151)		(0.104)
Log(electric)	0.417^***^		0.460^***^	
	(0.023)		(0.021)	
Age of Head	0.000	0.023^***^	−0.005^*^	0.038^***^
	(0.003)	(0.005)	(0.003)	(0.004)
Square of head’s age	−0.000	−0.000^***^	0.000	−0.000^***^
	(0.000)	(0.000)	(0.000)	(0.000)
Head is female	0.004	0.042	−0.018	0.071^***^
	(0.018)	(0.029)	(0.014)	(0.021)
Primary school	−0.013	0.153^***^	−0.005	0.118^***^
	(0.019)	(0.032)	(0.017)	(0.025)
Lower Secondary School	−0.029	0.254^***^	−0.018	0.255^***^
	(0.021)	(0.036)	(0.018)	(0.029)
Upper secondary school	0.017	0.312^***^	0.019	0.361^***^
	(0.027)	(0.053)	(0.023)	(0.040)
College or above	0.236^***^	0.117	0.242^***^	0.229^***^
	(0.035)	(0.091)	(0.028)	(0.063)
Head is married	−0.135^***^	0.290^***^	−0.105^***^	0.181^***^
	(0.041)	(0.064)	(0.032)	(0.048)
Head is widowed	−0.045	0.103	−0.038	0.052
	(0.043)	(0.068)	(0.035)	(0.052)
Head is divorced	−0.095^*^	0.207^**^	−0.009	0.075
	(0.057)	(0.090)	(0.041)	(0.060)
Head is separated	−0.143^*^	0.244^**^	0.038	−0.087
	(0.077)	(0.123)	(0.068)	(0.101)
The household has 1 child	−0.149^***^	0.184^***^	−0.088^***^	0.058^**^
	(0.017)	(0.033)	(0.015)	(0.025)
The household has 2 children	−0.256^***^	0.286^***^	−0.158^***^	0.064^*^
	(0.020)	(0.051)	(0.018)	(0.035)
The household has 3 or more children	−0.296^***^	0.249^***^	−0.247^***^	0.088
	(0.034)	(0.078)	(0.029)	(0.054)
The household has 1 elder	−0.101^***^	0.078^**^	−0.078^***^	−0.003
	(0.018)	(0.035)	(0.015)	(0.026)
The household has 2 or more elders	−0.127^***^	0.079^*^	−0.096^***^	0.003
	(0.025)	(0.047)	(0.018)	(0.031)
Number of members	−0.184^***^	0.384^***^	−0.300^***^	0.504^***^
	(0.019)	(0.027)	(0.017)	(0.025)
Square of the number of family members	0.012^***^	−0.026^***^	0.022^***^	−0.038^***^
	(0.002)	(0.003)	(0.002)	(0.002)
Households in rural areas	−0.052^***^	−0.249^***^	−0.121^***^	−0.152^***^
	(0.020)	(0.051)	(0.014)	(0.036)
The family owns the house	−0.235^***^		−0.274^***^	
	(0.041)		(0.028)	
The total area of the living room/bedroom		0.003^***^		0.003^***^
		(0.000)		(0.000)
Tile roof		−0.162^***^		−0.221^***^
		(0.019)		(0.021)
Sheet roof		−0.044^***^		−0.198^***^
		(0.015)		(0.017)
Straw roof		−0.389^***^		−0.478^***^
		(0.049)		(0.069)
Another roof type		−0.322^**^		−0.548^***^
		(0.142)		(0.189)
Constant	7.050^***^	−5.117^***^	7.756^***^	−2.413^**^
	(0.136)	(1.345)	(0.119)	(1.029)
Observations	8,574	8,574	8,531	8,531
R-squared	0.215	0.282	0.390	0.464

Standard errors in parentheses

*** p<0.01,

** p<0.05,

* p<0.1

The economy of scale effect of several household members still prevails but with relatively higher estimated coefficients. In this model, the interaction variables between gender and marital status are not included because the addition, if any, makes the regression system of equations more complex and harder to interpret. Nevertheless, the results show that a household head who gets married or a household head who gets divorced will have a significantly higher amount of electricity use per year than a single household head. In addition, having a child or an older person in the family is negatively correlated with the household’s total annual income.

The coefficients of age confirm the inverted U-shaped relationship with the family’s total electricity expenditure. The effects of being in different regions in the 3SLS model are lower than those in the OLS model. The exclusive exogenous variables, which play the role of instrumental variables, have statistically significant effects on income and electricity expenditure. Specifically, for electricity consumption, owning a house is associated with lower electricity consumption in a household. The estimated coefficient of electricity is statistically significant at a 1 per cent level in both 2012 and 2020 with very high t-statistics. This result shows that electricity impacts the family’s ability to earn income. The finding is similar to previous studies showing that electricity consumption improves household productivity, health status and education [3,37,48,50].

As presented in Table 6 above, we note that the total area of living/bedroom positively correlates with total electricity expenditure. The different roof types also explained the dispersion of electricity expenditure. Our empirical results also indicate that households will spend less money on electricity consumption if they own a house.

It should be noted that 3SLS is more sensitive to specification errors. Errors in one equation can propagate across the system, potentially affecting the overall estimation results. To mitigate the risk of misspecification, we have taken natural logarithms of both household electricity consumption and household income. We have also included squared terms for age and household size to capture the non-linear relationship between these variables and electricity expenditure, as supported by the current literature. For other explanatory variables, there is not much evidence in the current literature suggesting significant misspecification issues. Therefore, while we recognize the sensitivity of 3SLS, the careful design of our model partially ensures reliability of our findings.

## 5. Conclusions and policy implications

The world will continue facing challenges from ongoing economic crises, pandemics, climate changes, and political conflicts in the coming years. As a result, global energy security has become one of the main concerns of every government worldwide. Managing energy demand in general, and electricity demand in particular is urgent and necessary for any country, especially a developing country with high income and energy consumption growth like Vietnam. In Vietnam, household energy consumption continues to increase rapidly, reaching an average consumption per capita of 1,459 kWh/year in 2020 [57]. Specifically, the intensity of energy use in Vietnam over the past decade has reached nearly 1.5–2.0 times the GDP growth rate [19]. However, while electricity consumption is proliferating, the electricity supply and distribution infrastructure still need to be adequately developed. If these trends continue, Vietnam will increasingly rely on imported electricity and national energy security will not be guaranteed. As such, policies are urgently needed to promote the saving and efficient use of electricity for the household sector.

This study examines one of the most important aspects of household energy demand sources: the socio-demographic characteristics. The study uses two cross-sectional datasets from the Vietnamese Household Living Standard Surveys in 2012 and 2020 to understand the main socio-demographic factors influencing household expenditure on electricity and to identify the changes in the electricity consumption characteristics of Vietnamese households over the past decade. We note a sharp increase in the average household income in 2020 compared to 2012, implying a significant improvement in the standard of living of Vietnamese households during the 2012–2020 period. In addition, a positive relationship exists between a household’s income and the household’s electricity expenditure and between the household’s income and the educational attainment of the household’s head. In particular, the electricity consumption of the highest income group has increased sharply compared to the other income groups in 2020. The inverted U-shaped relationship between household size and electricity consumption is also confirmed, implying the economies of scale regarding household electricity consumption across Vietnamese households.

Key findings from our analysis identify three main factors leading to a basic understanding of the socio-demographic determinants of household electricity consumption across Vietnamese households. These factors include (i) the annual household income, (ii) the household size, and (iii) the education level of the household head.

Regarding household income, this study confirms a strong association between electricity consumption and household income. Specifically, households in the highest income decile spent nearly six times more on electricity consumption than those in the lowest income decile in 2012 and 2020. This can be due to the fact that higher-income households tend to live in larger homes with additional rooms and amenities, increasing energy demands for heating, cooling, and lighting. They may also own second homes, adding to total energy use. Additionally, as income rises, households often shift from traditional energy sources to electricity, preferring its convenience and efficiency for activities like cooking and heating. This transition aligns with the “energy ladder” hypothesis. Moreover, while high-income households may invest in energy-efficient appliances, the “rebound effect” can lead to higher usage, offsetting energy savings. Indeed, Zheng et al. [31] find that energy efficiency declines fastest in high-income groups, while low-income groups exhibit slower declines in efficiency. This finding indicates that the increasing block tariff schedule needs to be more effective and encourage high-income households to save electricity. In particular, the current wholesale electricity tariff in Vietnam includes four customer groups: (i) the wholesale electricity tariff for rural areas; (ii) the wholesale electricity tariff for housing blocks and residential clusters; (iii) the wholesale electricity tariff for commerce – service – residential building complex; and (iv) the wholesale electricity tariff for the industrial zone. Among these four groups, almost 60 different tariffs are applied to different tiers of consumers, depending on the amount of electricity consumption and different usage times, including standard, off-peak or peak hours. Regardless of the detailed structure, the difference among these tariff levels is significantly small, within the magnitude of less than VND 1,000 per kWh. For example, the tariff of VND 1.506 per kWh applies to the lowest group of consumers (Rate 1 for those using less than 50 kWh), whereas the tariff of VND 2,492 for kWh applies to Rate 6 (the highest group with the usage of more than 401 kWh). This small difference has failed to send clearer signals to electricity users with high incomes to save electricity because the budget for electricity consumption is too small in their household income and budget.

Education also has a major impact on household electricity consumption. People with a high level of education are more likely to have higher incomes and, therefore, be able to use more energy. However, the results of the analysis show that when controlling for income level, education is still a factor that strongly influences household electricity consumption. This finding suggests that when the head of household has a high level of education, their families tend to use more modern technological devices that consume electricity [29]. In addition, household heads with higher level of education are also more likely to be aware of the environmental and health benefits of using electricity compared to traditional energy sources. Thus, they tend to choose cleaner energy sources, like electricity, over traditional options such as biomass or coal [29,55]. As a result, households with higher education levels are more likely to use electricity for cooking, heating, and lighting. Although this shift supports cleaner energy use, it also leads to higher overall electricity consumption. This finding indicates that when the head of household has a high level of education, their families tend to use more modern technological devices that consume electricity and use cleaner energy sources, such as electricity, instead of traditional energy sources. This finding aligns well with those reported by Son and Yoon [29].

For other demographic factors, female-headed households use more energy than male-headed households. This can be explained by the view that many of the activities associated with electricity requirements, such as cooking and washing, are carried out by women [56]. The scale effect of household size occurs in the Vietnamese context. Total electricity consumption does not continue to increase as household size increases but starts to decrease after household size reaches a certain point where family members can share the use of electrical appliances. The inverted U-shaped relationship between the age of the household head and their family’s electricity expenditure is also observed in the results. We also find that households in the Red River Delta and Southeast regions spend more electricity than others. Compared to 2012, households living in the Red River Delta region in 2020 have a considerable increase in average electricity expenditure compared to other regions. This is mainly because of the following reasons: First, the Red River Delta is a densely populated area, where Ha Noi, the capital of Vietnam, is located [58]. Second, in recent years, under the influence of climate change, Vietnam has experienced heat waves with increasing frequency and intensity, which have negatively impacted people’s lives. The effects of heat waves are particularly evident in the Red Delta region of Vietnam [59]. Finally, the high speed of urbanization also leads to significant temporal changes in the land surface temperature of the urban area [60]. These reasons eventually increase the need for electrical equipment for heating and cooling and result in higher electricity consumption.

Our findings also indicate that the house’s size has a positive correlation with electricity consumption, but the magnitude of this relationship is very small. In addition, house types do have a strong correlation with the total electricity expenditure of a household. We find that families living in temporary houses spend much less money on electricity than those living in permanent houses. When different homes, such as roof types and wall materials, are used, the relationships remain significant in 2012 and 2020. Finally, the average temperature of the province where a household resides is strongly correlated with electricity consumption.

These results suggest implications for policymakers in Vietnam in designing electricity demand-side management strategies and designing policies that align household electricity consumption patterns with broader climate change mitigation strategies. Household energy use contributes significantly to greenhouse gas emissions, particularly in developing nations like Vietnam, where energy infrastructure relies heavily on fossil fuels. Several policy implications have emerged. *First*, the findings from this study show that the increasing block tariffs need to be revised to focus more on high-income groups because the current tariff schedule is not efficient enough to affect the high-income groups’ electricity use behaviour. Policymakers should consider revising the increasing block tariffs scheme to introduce additional tiers with higher price increases for higher consumption levels. These measures promote energy saving, thereby contributing to emissions reductions, which is a fundamental aspect of global climate change mitigation strategies. *Second*, the growing disparity in electricity consumption between high and low income households, as evidenced by the sharp increase in consumption inequality in 2020, calls for policies to protect disadvantaged households while addressing the environmental impacts of energy use. Given the significant gap in electricity expenditure between high and low income households, targeted subsidy programs for low-income households should be given more attention. Current subsidies could be expanded to cover not only direct electricity costs but also energy-efficient appliances, such as LED lighting, energy-saving refrigerators, and efficient air conditioners. Policymakers may also consider providing financial support and/or low-interest loans for purchasing energy-efficient appliances, which can enable poorer households to reduce their electricity bills while improving their standard of living. This approach would also help narrow the disparity in energy consumption across income groups and support energy security and environmental sustainability. In addition, subsidy programs targeting poor households should be given more attention to reducing energy consumption dispersion. *Third*, the government may need to implement various policies targeting income inequality in Vietnam, thereby reducing inequality in energy consumption. Policymakers should prioritize policies aimed at reducing income inequality, such as progressive taxation, improved access to education and job training, and social safety nets for low-income families. *Fourth*, our findings indicate that households with heads who have a higher level of education tend to have higher electricity consumption, implying that they and their family members are more likely to adopt new technology and use more electrical appliances. As such, energy-saving policies should also aim at adults, rather than just educating children. Policymakers may consider implementing comprehensive public awareness campaigns that emphasize the importance of energy conservation and provide practical experience on reducing electricity usage, targeting not just children in schools but also adult consumers. In addition, as most educated households are employed, governments may encourage and offer incentives for companies and organizations that provide their employees with workshops, training, and/or financial support for purchasing energy-efficient home appliances. In the broader context of climate change mitigation, our results emphasize the need to consider socio-economic factors, rather than solely technological factors and energy prices, when designing energy policies that aim to reduce emissions through demand-side management strategies. This insight is particularly relevant for climate change mitigation, as it suggests that a one-size-fits-all approach to policy may not be as effective as context-sensitive ones.

This study exhibits various limitations. The study focuses on only two waves of the VHLSS surveys in 2012 and 2020. Given the dynamic nature of the households’ finances in Vietnam after the COVID-19 pandemic, including information from the latest VHLSS survey in 2022 (after the pandemic), albeit unavailable at the time this study was conducted, is highly desirable. In addition, this study has only focused on two waves of the VHLSS surveys in 2012 and 2020, so the important information embedded in other waves of the VHLSS may need to be better captured in the results presented in this study. As such, future studies may need to incorporate the latest data once available for VHLSS 2022 and all other waves of VHLSS surveys by tracking the households participating in various VHLSS surveys. Besides, the reliance on cross-sectional data is somehow less efficient than longitudinal data in fully capturing individual household dynamics over time. Future research could address these limitations by incorporating additional waves of VHLSS data or employing longitudinal datasets to better understand the longitudinal impact of socio-demographic changes on household energy use. This study also utilizes available data from the VHLSS surveys, which need to include responses regarding households’ lifestyles and consumption habits. We consider that the households’ lifestyles and consumption habits may significantly affect the choice of electricity consumption. For instance, households with members who work remotely or spend more time at home generally experience higher electricity consumption due to the increased use of appliances such as computers, air conditioners, and lighting during the day. On the other hand, households with individuals working in jobs that require extensive travel or long hours away from home may have lower electricity usage. In addition, consumption habits can also influence electricity consumption. For example, households that adopt energy-efficient appliances and incorporate renewable energy sources, such as solar panels, often face lower utility bills. Conversely, households with habits of using high-energy appliances, such as large refrigerators, electric heaters, or dryers, without moderation tend to face higher electricity bills. Besides, lifestyle and consumption habits may also affect household income through health and productivity impacts. As such, this important aspect should be explored in future studies.

## Appendix: Relevant questions regarding the household incomes in the VHLSS surveys

### I. Labour incomes:

1. How much in salaries/wages, including that in kind, has [name] received from this job over the last 12 months? (1000 VND)
2. Over the past 12 months, how much in cash and kind, apart from salaries/wages from this job, has [name] received from the following: (1000 VND) a) Festive occasions (1/5, 2/9, 22/12, Lunar, etc.) and b) Others (bonuses, uniforms, lunch, allowances for business, trips, sickness, labor accidents, pregnancy...)
3. How much in salaries/wages, including that in kind, has [name] received from this job over the last 12 months? (1000 VND)
4. Over the past 12 months, how much in cash and kind, apart from salaries/wages from this job, has [name] received from the following: (1000 VND) a) Festive occasions (1/5, 2/9, 22/12, Lunar, etc.) and b) Others (bonuses, uniforms, lunch, allowances for business, trips, sickness, labor accidents, pregnancy...)
5. Apart from the foregoing jobs, has [name] taken any other salaried/waged jobs? And how much money has [name] received from these jobs? (from the third job onwards) (1000 VND)
6. Has [name] received unemployment benefits, one-off severance pays, pensions, allowance for loss of working capacity over the past 12 months? And how much has he/she received over the past 12 months? (1000 VND)

### II. In come from other household activities

#### 1. Farm, forestry, and aquaculture activities.

Cultivation
  Rice: What are the proceeds from sales or barter of [Rice] over the last 12 months? (1000 VND)
  Staple food crops, non-staple food crops, and other annual crops: The total revenues from sale or barter of [Kind] over the last 12 months? (1000 VND)
  Annual and perennial industrial crops: What are the proceeds from sales or barter of [Kind] over the last 12 months? (1000 VND)
  Fruit trees: The total revenues from sales or barter of [Fruit trees] over the last 12 months? (1000 VND)
  Revenues from harvested by-products and collected products: What are the revenues from sales of [Kind] over the last 12 months? (1000 VND)
Animal husbandry and hunting, trapping, domestication *of* birds and animals
  What is the value of the output obtained over the last 12 months? (1000 VND)
Agricultural services
  a) How many months of activities over the last 12 months?
  b) How much have you earned per month on average? (1000 VND)
  c) Total revenues (1000 VND) = (a) * (b)
Forestry
  Values of outputs/revenues from activities over the past 12 months? (1000 VND)
Aquaculture
  Total values of products gained over the past 12 months (1000 VND)
Domains *of* production and business, non-agricultural, forestry and aquaculture services; processing *of* agricultural, forestry and aquatic products
  Revenue over the past 12 months? (1000 VND)

#### 2. Other revenues included in incomes.

Has anyone in your household, over the past 12 months, received cash or kind from the following sources? And value of each source over the past 12 months.

a) Cash and kind sent as a gift or aid for domestic use by non-members of the household from overseas.
b) Cash and kind (value) for domestic use sent as a gift or aid by relatives residing and working overseas temporarily.
c) Gift of housing
d) Gift of automobile(s) for domestic use
e) Other gifts of assets for domestic use
f) Wedding cash gifts after deducting expenses of guests’ food and drinks.
g) Funeral cash tributes after deducting expenses of guests’ food and drinks.
h) Social benefits for war invalids, families of fallen combatants, and individuals/families with revolutionary merits.
i) Social benefits for beneficiary households of social policies
j) Assistance to overcome natural disasters and fire.
k) From types of insurance (excluding social, health and life insurance)
l) Interests of savings deposits, stocks, shares, lending, contributed capital.
m) Revenues from renting out workshop floors, machines, assets, and facilities not included in sections of sectoral production and business (except housing, farming and forest land, and water surface for aquaculture production)
n) Revenues as donations from organizations, humanitarian aid, associations and units of production and business
o) Others (Specify_____)

### III. Housing

Does your household receive rents from those residential land lots or houses?
How much has your household received from leasing residential land and houses over the past 12 months?

## References

[1] Balat M. Electricity from worldwide energy sources. Energy Sources, Part B: Econ Plann Policy. 2006;1(1):395–412.

[2] Winter C-J. Electricity, hydrogen—Competitors, partners? Int J Hydr Energy. 2005;30:1371–4.

[3] Bridge BA, Adhikari D, Fontenla M. Electricity, income, and quality of life. Soc Sci J. 2016;53:33–9.

[4] Khandker SR, Barnes DF, Samad HA, Minh NH. Welfare Impacts of Rural Electrification: Evidence from Vietnam [SSRN Scholarly Paper]. Rochester, NY: 2009. Available from: https://papers.ssrn.com/abstract=1476699

[5] Hu Z, Wang M, Cheng Z. Mapping the knowledge development and trend of household energy consumption. Environ Dev Sustain. 2022;24:6053–71.

[6] Waheed R, Sarwar S, Wei C. The survey of economic growth, energy consumption and carbon emission. Energy Rep. 2019;5:1103–15. doi: 10.1016/j.egyr.2019.01.001

[7] Wang X, Feng W, Cai W, Ren H, Ding C, Zhou N. Do residential building energy efficiency standards reduce energy consumption in China? – A data-driven method to validate the actual performance of building energy efficiency standards. Energy Policy. 2019;131:82–98.

[8] Kaufmann RK, Gopal S, Tang X, Raciti SM, Lyons PE, Geron N, et al. Revisiting the weather effect on energy consumption: implications for the impact of climate change. Energy Policy. 2013;62:1377–84.

[9] Iyke BN. Climate change, energy security risk, and clean energy investment. Energy Econ. 2024;129:107225. doi: 10.1016/j.eneco.2024.107225

[10] Ma B, Sharif A, Bashir M, Bashir MF. The dynamic influence of energy consumption, fiscal policy and green innovation on environmental degradation in BRICST economies. Energy Policy. 2023;183:113823.

[11] Tanveer A, Song H, Faheem M, Daud A. The paradigms of transport energy consumption and technological innovation as a panacea for sustainable environment: is there any asymmetric association? Environ Sci Pollut Res Int. 2023;30(8):20469–89. doi: 10.1007/s11356-022-23453-3 36255583

[12] Tanveer A, Song H, Faheem M, Daud A, Naseer S. Unveiling the asymmetric impact of energy consumption on environmental mitigation in the manufacturing sector of Pakistan. Environ Sci Pollut Res Int. 2021;28(45):64586–605. doi: 10.1007/s11356-021-14955-7 34318417

[13] Tanveer A, Song H, Faheem M, Daud A, Safdar N. Navigating the asymmetric influence of financial inclusion on environmental sustainability: dynamic role of energy consumption and human capital. Energy Environ. 2024;35(6):3087–115. doi: insert_doi_here

[14] Ibrahim RL, Ajide KB, Omokanmi OJ. Non-renewable energy consumption and quality of life: evidence from Sub-Saharan African economies. Resourc Policy. 2021;73:102176.

[15] Mišík M. The EU needs to improve its external energy security. Energy Policy. 2022;165:112930.

[16] Daioglou V, van Ruijven BJ, van Vuuren DP. Model projections for household energy use in developing countries. Energy. 2012;37:601–15. doi: 10.1016/j.energy.2011.10.020

[17] Reid L, Sutton P, Hunter C. Theorizing the meso level: the household as a crucible of pro-environmental behaviour. Progress Hum Geogr. 2010;34:309–27.

[18] Han X, Wei C. Household energy consumption: State of the art, research gaps, and future prospects. Environ Dev Sustain. 2021;23:12479–504. doi: 10.1007/s10668-021-01283-5

[19] Le PV. Energy demand and factor substitution in Vietnam: evidence from two recent enterprise surveys. J Econ Struct. 2019;8:35.

[20] Kikuchi T, Yanagida K, Vo H. The effects of Mega-Regional Trade Agreements on Vietnam. J Asian Econ. 2018;55(1):4–19.

[21] World Economic Forum (2023). Vietnam’s $135 billion power plan for 2030. Available from: https://www.weforum.org/agenda/2023/05/vietnam-pdp8-power-plan-for-2030.

[22] EVN. Annual Report 2021. 2021. [cited Aug 30 2022]. Available from: https://en.evn.com.vn/d6/news/Annual-Report-2021-6-13-2537.aspx

[23] Costanzo M, Archer D, Aronson E, Pettigrew T. Energy conservation behaviour: the difficult path from information to action. Am Psychol. 1986;41:521–8. doi: 10.1037/0003-066X.41.6.521

[24] Abrahamse W, Steg L. How do socio-demographic and psychological factors relate to households’ direct and indirect energy use and savings? J Econ Psychol. 2009;30(5):711–20. doi: 10.1016/j.joep.2009.05.006

[25] Bhattacharjee S, Reichard G. Socio-Economic Factors Affecting Individual Household Energy Consumption: A Systematic Review. 2012; 891–901. American Society of Mechanical Engineers Digital Collection.

[26] Brandon G, Lewis A. Reducing household energy consumption: a qualitative and quantitative field study. J Environ Psychol. 1999;19:75–85.

[27] Brounen D, Kok N, Quigley JM. Residential energy use and conservation: economics and demographics. Eur Econ Rev. 2012;56(5):931–45. doi: 10.1016/j.euroecorev.2012.02.007

[28] Van Raaij WF, Verhallen TMM. A behavioural model of residential energy use. J Econ Psychol. 1983;3:39–63.

[29] Son H, Yoon S. Reducing energy poverty: Characteristics of household electricity use in Vietnam. Energy Sustain Dev. 2020;59:62–70.

[30] Nguyen H.-S. Exploring the determinants of household electricity demand in Vietnam in the period 2012–16 (PhD thesis, Université Paris Saclay (COmUE)). Université Paris Saclay (COmUE); 2019.

[31] Zheng J, Dang Y, Assad U. Household energy consumption, energy efficiency, and household income–evidence from China. Appl Energy. 2024;353:122074.

[32] Vo DH, Vo LH, Ho CM. Regional convergence of nonrenewable energy consumption in Vietnam. Energy Policy. 2022;169:113194.

[33] Vo DH, Ho CM, Vo AT. Trade openness, financial development, and urbanization in the renewable energy-growth-environment nexus. Energy Sources, Part B: Econ Plann Policy. 2023;18(1):2240784.

[34] Ekholm T, Krey V, Pachauri S, Riahi K. Determinants of household energy consumption in India. Energy Policy. 2010;38:5696–707.

[35] Kostakis I. Socio-demographic determinants of household electricity consumption: Evidence from Greece using quantile regression analysis. Curr Res Environ Sustain. 2020;1:23–30.

[36] Rahut DB, Das S, De Groote H, Behera B. Determinants of household energy use in Bhutan. Energy. 2014;69:661–72.

[37] Bridge BA, Adhikari D, Fontenla M. Household-level effects of electricity on income. Energy Econ. 2016;58:222–8.

[38] Özcan KM, Gülay E, Üçdoğruk Ş. Economic and demographic determinants of household energy use in Turkey. Energy Policy. 2013;60:550–7.

[39] Yarbaşı İY, Çelik AK. The determinants of household electricity demand in Turkey: An implementation of the Heckman Sample Selection model. Energy. 2023;283:128431.

[40] Miles D. A household level study of the determinants of incomes and consumption. Econ J. 1997;107(1):1–25.

[41] Zou B, Luo B. Rural household energy consumption characteristics and determinants in China. Energy. 2019;182:814–23. doi: 10.1016/j.energy.2019.06.048

[42] Cheng C, Liu Y, Han C, Fang Q, Cui F, Li X. Effects of extreme temperature events on deaths and its interaction with air pollution. Sci Total Environ. 2024;915:170212. doi: 10.1016/j.scitotenv.2024.170212 38246371

[43] Han Y, Du X, Zhang H, Ni J, Fan F. Does smart home adoption reduce household electricity-related CO2 emissions?–Evidence from Hangzhou city, China. Energy. 2024;289:129890.

[44] Mizobuchi K, Hiroaki Y. Impact of time-saving technology on household electricity consumption: an automatic vacuum cleaner distribution experiment in Japan. Ecol Econ. 2024;223:108231. doi: 10.1016/j.ecolecon.2024.108231

[45] Schleich J, Schuler J, Pfaff M, Frank R. Do green electricity tariffs increase household electricity consumption? Appl Econ. 2022;55(20):2337–48. doi: 10.1080/00036846.2022.2102574

[46] Wang Y, Hou L, Cai W, Zhou Z, Bian J. Exploring the drivers and influencing mechanisms of urban household electricity consumption in China-based on longitudinal data at the provincial level. Energy. 2023;273:127191.

[47] Frederiks ER, Stenner K, Hobman EV. The socio-demographic and psychological predictors of residential energy consumption: a comprehensive review. Energies. 2015;8(5):573–609. doi: 10.3390/en8050573

[48] Khandker SR, Barnes DF, Samad HA. Welfare impacts of rural electrification: a panel data analysis from Vietnam. Econ Dev Cult Change. 2013;61(4):659–92.

[49] Phung DT, Nguyen P. Vietnam Household Living Standard Surveys 2002 and 2004: Basic Information. GSOVN: 2007.

[50] Khandker S, Barnes D, Samad H. Are the energy-poor also income poor? Evidence from India. Energy Policy. 2012;47:1–12.

[51] Zellner A. An efficient method of estimating seemingly unrelated regressions and tests for aggregation bias. J Am Stat Assoc. 2012;57(298):348–68.

[52] Belderbos R, Leten B, Nguyen NH, Vancauteren M. Multinational firms and the quest for global talent: employing (skilled) foreign workers at home and abroad. J Int Bus Stud. 2023:1–23.

[53] Soda G, Stea D, Pedersen T. Network structure, collaborative context, and individual creativity. J Manage. 2019;45(4):1739–65.

[54] Ironmonger DS, Aitken CK, Erbas B. Economies of scale in energy use in adult-only households. Energy Econ. 1995;17(3):301–10.

[55] Vo DH, Vo AT, Ho CM. Understanding the characteristics of the household energy transition in a developing country. Heliyon. 2024;10(1):e23977. doi: 10.1016/j.heliyon.2024.e23977 38234912 PMC10792559

[56] Grünewald P, Diakonova M. Societal differences, activities, and performance: Examining the role of gender in electricity demand in the United Kingdom. Energy Res Soc Sci. 2020;69:101719. doi: 10.1016/j.erss.2020.101719

[57] World Data. Energy consumption in Vietnam. 2022. [cited August 11 2022]. Available from: https://www.worlddata.info/asia/vietnam/energy-consumption.php.

[58] Binh TQ, Phuong PT, Nhung BT, Tung DD. Metabolic syndrome among a middle-aged population in the Red River Delta region of Vietnam. BMC Endocr Disord. 2014;14:77. doi: 10.1186/1472-6823-14-77 25261978 PMC4179436

[59] Tuyet MTT, Van Vo H, Le Danh T. Application of self-organizing maps and K–Means methods to classify summer heat wave weather patterns in Viet Nam. Vietnam J Hydrometeorol. 2022;11:15–25.

[60] Van Nguyen O, Kawamura K, Trong DP, Gong Z, Suwandana E. Temporal change and its spatial variety on land surface temperature and land use changes in the Red River Delta, Vietnam, using MODIS time-series imagery. Environ Monit Assess. 2015;187(7):464. doi: 10.1007/s10661-015-4691-3 26113204

